# Co-reductive fabrication of carbon nanodots with high quantum yield for bioimaging of bacteria

**DOI:** 10.3762/bjnano.9.16

**Published:** 2018-01-12

**Authors:** Jiajun Wang, Xia Liu, Gesmi Milcovich, Tzu-Yu Chen, Edel Durack, Sarah Mallen, Yongming Ruan, Xuexiang Weng, Sarah P Hudson

**Affiliations:** 1 College of Chemistry and Life Science, Zhejiang Normal University, Jinhua 321004, PR China; 2 Department of Chemical Sciences, Bernal Institute, University of Limerick, Castletroy, Ireland; 3 School of Chemistry, University of Birmingham, Edgbaston, Birmingham B15 2TT, UK

**Keywords:** bioimaging, carbon nanodots, collaborative reduction, hydrothermal

## Abstract

A simple and straightforward synthetic approach for carbon nanodots (C-dots) is proposed. The strategy is based on a one-step hydrothermal chemical reduction with thiourea and urea, leading to high quantum yield C-dots. The obtained C-dots are well-dispersed with a uniform size and a graphite-like structure. A synergistic reduction mechanism was investigated using Fourier transform infrared spectroscopy and X-ray photoelectron spectroscopy. The findings show that using both thiourea and urea during the one-pot synthesis enhances the luminescence of the generated C-dots. Moreover, the prepared C-dots have a high distribution of functional groups on their surface. In this work, C-dots proved to be a suitable nanomaterial for imaging of bacteria and exhibit potential for application in bioimaging thanks to their low cytotoxicity.

## Introduction

Over recent years, carbon nanomaterials have remarkably influenced the growth of a wide range of fields, including electronics, photonics, energy, catalysis and medicine. Within this class of materials, carbon nanodots (C-dots) are deemed a major breakthrough for the development of fluorescent nanomaterials. They are a promising alternative to fluorescent inorganic semiconductor nanocrystals and organic dyes due to their chemical stability, good dispersibility in water, low photobleaching and low cytotoxicity. They show great potential for bioimaging, photocatalysis, energy conversion, fluorescent ink and sensing applications [1–3]. In a bioimaging application perspective, the detection of bacteria by microscopic visualization is an essential benchmark. Currently, visual detection approaches are based on indirect methods related to bacterially secreted metabolites or imaging of bacterial colonies [4]. Furthermore, the staining techniques use either commercially available fluorescent dyes or semiconductor quantum dots [5]. Fluorescent dyes are expensive, instable and easily susceptible to photobleaching, while the semiconductor quantum dots are toxic and difficult to dissolve in water. Therefore, simple and inexpensive methods to visualize the morphological details of bacterial cells are highly needed.

C-dots can thus be proposed as an innovative platform for bioimaging purposes thanks to their fluorescent features. In this context, the quantum yield (QY) is one of the most important features for C-dot performance. Although, at present, the actual mechanism of the photoluminescence of C-dots is still an open debate among researchers [6–8], significant progress in increasing the QY has been achieved. Most of the mentioned methods refer to surface passivation [9–11] and doping [12–13]. Recently, chemical reduction was also reported as an effective method to enhance the QY of C-dots [14]. Zheng et al. found an increase in QY for C-dots from 2% to 24% following reduction with sodium borohydride (NaBH_4_). The same results were confirmed by Shen and Tian's group [15–16]. It was also reported that the fluorescence intensity of graphene quantum dots reduced by hydrazine hydrate (N_2_H_4_) can be enhanced to more than two times that of the pristine graphene quantum dots [17]. However, this reduction pathway is based on a two-step procedure: firstly, a synthesis and collection of bare C-dots, then a reduction of C-dots to enhance their QY. The above procedure is often time consuming, poses difficulty in achieving a final pure sample, and introduces secondary pollution products. Therefore, in order to promote and extend their range of applications, new methods to obtain C-dots with high QY are required.

Citric acid, citrate, urea or thiourea have been used in the past to obtain high-QY C-dots with different growth mechanisms proposed [18–20]. Qu et al. obtained graphene quantum dots with a quantum yield of 78% and 71% using citric acid and urea or citric acid and thiourea as the precursors, respectively. They demonstrated that N or N/S doping led to the high QY of the C-dots. Zeng et al. prepared C-dots with a relatively high QY value (45%) using citric acid and urea as precursors via a facile hydrothermal method. They evidenced that surface passivation by urea resulted in the high QY of the C-dots. Herein we report a C-dot synthetic procedure with remarkable QY (37%) by a one-step hydrothermal chemical reduction method, where sodium citrate is the carbon source and urea and thiourea are the co-reducers. Specifically, the amount of citrate was kept constant and the molar ratio of urea and thiourea were varied to demonstrate the effects of thiourea and urea on the different QYs. The results showed C-dots prepared with both urea and thiourea present more reduced carbon and exhibit a higher QY under the synergistic reduction way. Compared with a conventional two-step chemical reduction pathway, the one-step method is efficient and eco-friendly. Moreover, the obtained C-dots with abundant functional groups on the C-dot surface and high QY exhibit excellent potential for use as bacteria (*Xanthomonas axonopodis* pv. *glycins*, *Xag*) imaging agents.

## Results and Discussion

### Synthesis of carbon nanodots

As shown in Table 1, the C-dots with different QYs were obtained as the amount of sodium citrate was kept constant and the molar ratio of urea and thiourea was varied. Remarkably, C-dots from sodium citrate, urea and thiourea resulted in a higher QY than those of citrate and urea or citrate and thiourea. To explain the differences in QY for these samples, we propose that sodium citrate serves as a self-assembly trigger for a carbon-based structure due to the intermolecular H-bonding. Subsequently, a condensation process takes place, forming C-dots. Meanwhile, the gradual, homogenous release of OH^−^ and NH_3_ from urea hydrolysis [21] and H_2_S from thiourea led to the formation of C-dots under alkaline, reducing and hydrothermal conditions [22–25]. Therefore, C-dots prepared with both urea and thiourea present a higher amount of reduced carbon and exhibit a higher QY under the co-reduction pathway. It was also found that increasing the thiourea concentration above 0.014 M during the synthesis process resulted in a gradual decrease in QY. The results indicated that it acquired a highest reduced atmosphere when the molar ratio of urea to thiourea is about 3.

**Table 1:** Fluorescent carbon nanodots with different quantum yields synthesized using different additives.

Sample label^a^	Sodium citrate (mmol)	Urea (mmol)	Thiourea (mmol)	QY (%)

S_a_	0.28	1.68	0	8
S_b_	0.28	1.26	0.42	37
S_c_	0.28	0.84	0.84	20
S_d_	0.28	0.42	1.26	14
S_e_	0.28	0	1.68	2

^a^All the samples were dissolved in 30 mL of water.

To demonstrate the rationale of co-reduced C-dot production method, glucose and xylose were used as the model carbon source references. Glucose and xylose were selected due to their broad range of use for C-dot fabrication [26–27] and the obtained QYs are shown in Supporting Information File 1,Table S1. The results further prove that mixed reducing conditions can enhance the QY, irrespective of the carbon source used.

Due to the wide range of C-dot applications, their quantitative yield for mass production must be improved. As shown in Supporting Information File 1,Table S2, 4.0 to 5.0 g of C-dots were prepared by increasing the concentration of the reagents, while still keeping the molar ratios constant during the synthesis. It was found that the QY of the C-dots with co-reduction using urea and thiourea is much higher than when urea or thiourea are used individually during reduction. Moreover, during the scale-up synthesis, it was determined that the C-dots tend to aggregate due to their larger magnitude in mass, leading to an increase in the size of the synthesized C-dots (Figure S1, Supporting Information File 1) and a decrease in the QY.

### Characterization of the carbon nanodots

**Figure 1:**
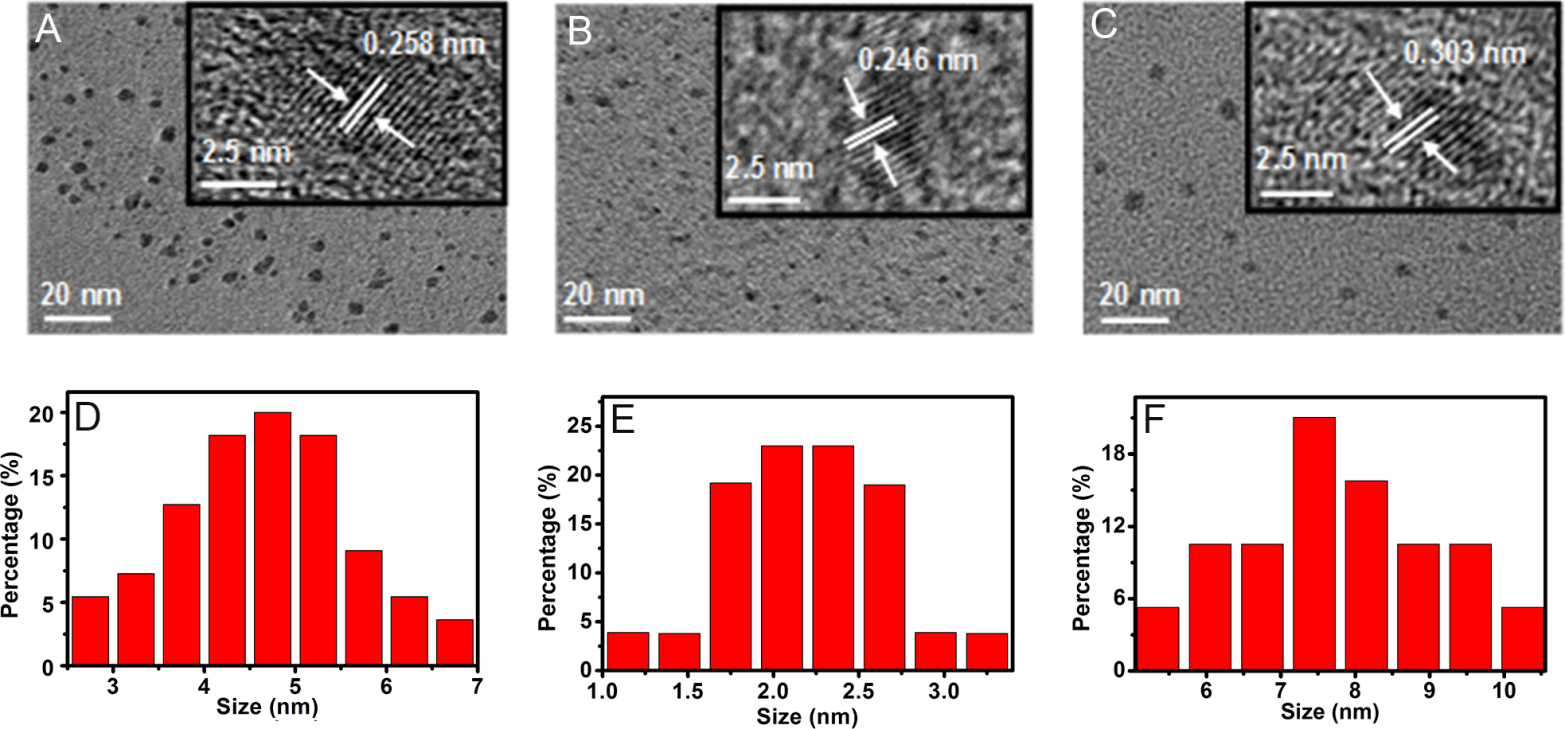
TEM and HRTEM (inset) images of (A) S_a_, (B) S_b_, (C) S_e_ samples, and corresponding size (diameter) distribution ranges for (D) S_a_, (E) S_b_, and (F) S_e._

The morphology of the products was characterized by transmission electron microscopy (TEM) and high-resolution transmission electron microscopy (HRTEM). Figure 1A–C shows the TEM images of the S_a_, S_b_ and S_e_ samples. As it can be observed, all the C-dots are consistently dispersed and separated from each other. The three samples show lattice fringes with distances of 0.258 (inset in Figure 1A), 0.246 (inset in Figure 1B) and 0.303 nm (inset in Figure 1C), respectively. These are consistent with the (102), (100) and (002) diffraction planes, respectively, of sp^2^ graphitic carbon [28–29]. The corresponding particle size distribution histograms (Figure 1D–F) show the average diameter of the S_a_, S_b_ and S_e_ materials is 4.7 ± 1.0 nm, 2.2 ± 0.5 nm and 7.8 ± 1.8 nm, respectively.

X-ray diffraction (XRD) was used to investigate the crystallinity of S_a_, S_b_ and S_e_. As shown in Figure S2 in Supporting Information File 1, sample S_a_ and S_e_ display a broad diffraction peak centered at around 22.7°, which is similar to the (002) lattice spacing for graphitic (sp^2^) carbon [30–33]. However, for the pattern of S_b_, the peak at 22.7° is much stronger and an extra peak centered at 15.9° appears, which refers to the (103) planes of hexagonal carbon [9]. It can be noticed that the crystallite size from the diffraction peaks does not perfectly match with the lattice spacing observed in TEM. This could be ascribed to the fact that the average polycrystalline signal is collected in XRD, while in TEM, only the single crystallite is investigated.

The mean crystallite size of the C-dots was estimated by using Scherrer’s equation, *D* = *k*λ/βcosθ, where *D* is the average crystallite size, *k* is a geometrical factor (0.89), λ is the wavelength of the monochromatic X-rays (Cu Kα radiation, λ = 1.5404 Å), θ is the Bragg angle and β is the full-width at half-maximum intensity of the diffraction peak (in radians) at 2θ [34]. The average crystallite size of S_a_, S_b_ and S_e_ calculated from the XRD patterns is 3.3, 2.85 and 3.9 nm, respectively, which is consistent with the size distribution ranges observed in TEM.

In order to further confirm the intrinsic carbogenic structure, the Raman spectra (λ_ex_ = 633 nm) of S_a_, S_b_ and S_e_ are shown in Figure S3, Supporting Information File 1. Two typical peaks for carbon can be clearly detected for S_a_, S_b_ and S_e_. The D-band, located at 1387 cm^−1^, correlates to the disorder or defects in the graphitized structure (sp^3^-hybridized carbon), while the G-band (1540 cm^−1^) is assigned to the E_2g_ mode of graphite and corresponds to the vibration of sp^2^-bonded carbon atoms in a two-dimensional hexagonal [35]. The intensity ratio of the D- to G-band (*I*_D_/*I*_G_) is a measure of the extent of disorder, and the ratio of sp^3^/sp^2^ carbon. S_a_ has an *I*_D_/*I*_G_ ratio of 0.86, and a ratio of 1 in both S_b_ and S_e_ was found. The lower *I*_D_/*I*_G_ value of S_b_ suggests that S_b_ is composed of more sp^2^-bonded carbon atoms [36], which agrees well with the proposed co-reduction pathway to effectively produce more reduced carbon with a higher C-dot QY.

**Figure 2:**
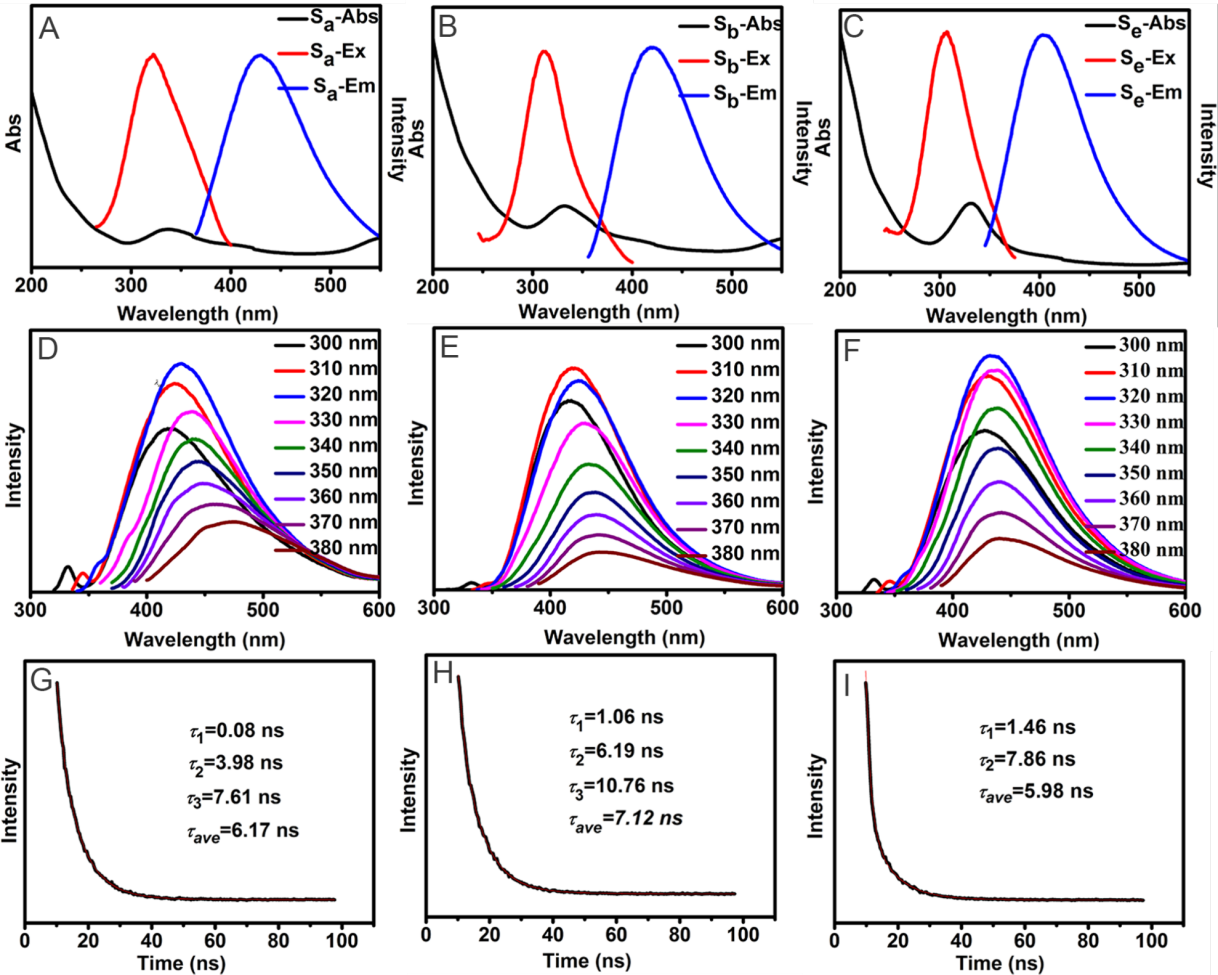
(A–C) UV–vis absorption, excitation and emission spectra, (D–F) fluorescence emission spectra recorded at different excitation wavelengths and (G–I) fluorescence intensity decay curves of S_a_, S_b_ and S_e_, respectively_._

UV–vis absorption and fluorescence properties were studied at room temperature to explore the optical properties of the three optimized C-dots. As shown in Figure 2A–C, UV–vis spectra of S_a_, S_b_ and S_e_ all show typical n→π* transition absorption peaks at 336 nm, 332 nm and 330 nm, respectively. S_a_, S_b_ and S_e_ display their highest emission intensity at 430 nm, 420 nm and 430 nm, corresponding to 320 nm, 310 nm and 320 nm excitation, respectively. The emission wavelengths of S_a_, S_b_ and S_e_ are dependent on the excitation wavelengths in the range of 300–380 nm, red-shifting with the gradual increase of the excitation wavelength (Figure 2D–F). Furthermore, the fluorescent decay dynamics for S_a_, S_b_ and S_e_ were also investigated (Figure 2G–I). The time-resolved decay curves of S_a_ and S_b_ are well fitted with a tri-exponential function, while that of S_e_ is fitted with a bi-exponential function. The fluorescence characteristic parameters, including the excitation (λ_ex_) and emission (λ_em_) wavelengths, quantum yield (Φ), lifetime components (τ_1_, τ_2_, τ_3_) and fluorescence lifetime (τ_ave_) for S_a_, S_b_ and S_e_ are listed in Table 2.

**Table 2:** The excitation (λ_ex_) and emission (λ_em_) wavelengths, quantum yield (Φ), lifetime components (τ_1_, τ_2_, τ_3_) and fluorescence lifetime (τ_ave_) for S_a_, S_b_ and S_e_.

	λ_ex_ (nm)	λ_em_ (nm)	Φ	τ_1_ (ns)	τ_2_ (ns)	τ_3_ (ns)	τ_ave_ (ns)

S_a_	320	430	8	0.08 (1.0%)	3.98 (19.4%)	7.61 (79.25%)	6.17
S_b_	310	420	37	1.06 (2.82%)	6.19 (73.68)	10.76 (23.5%)	7.12
S_e_	320	430	2	1.46 (29.3%)	7.86 (70.7%)	–	5.98

**Figure 3:**
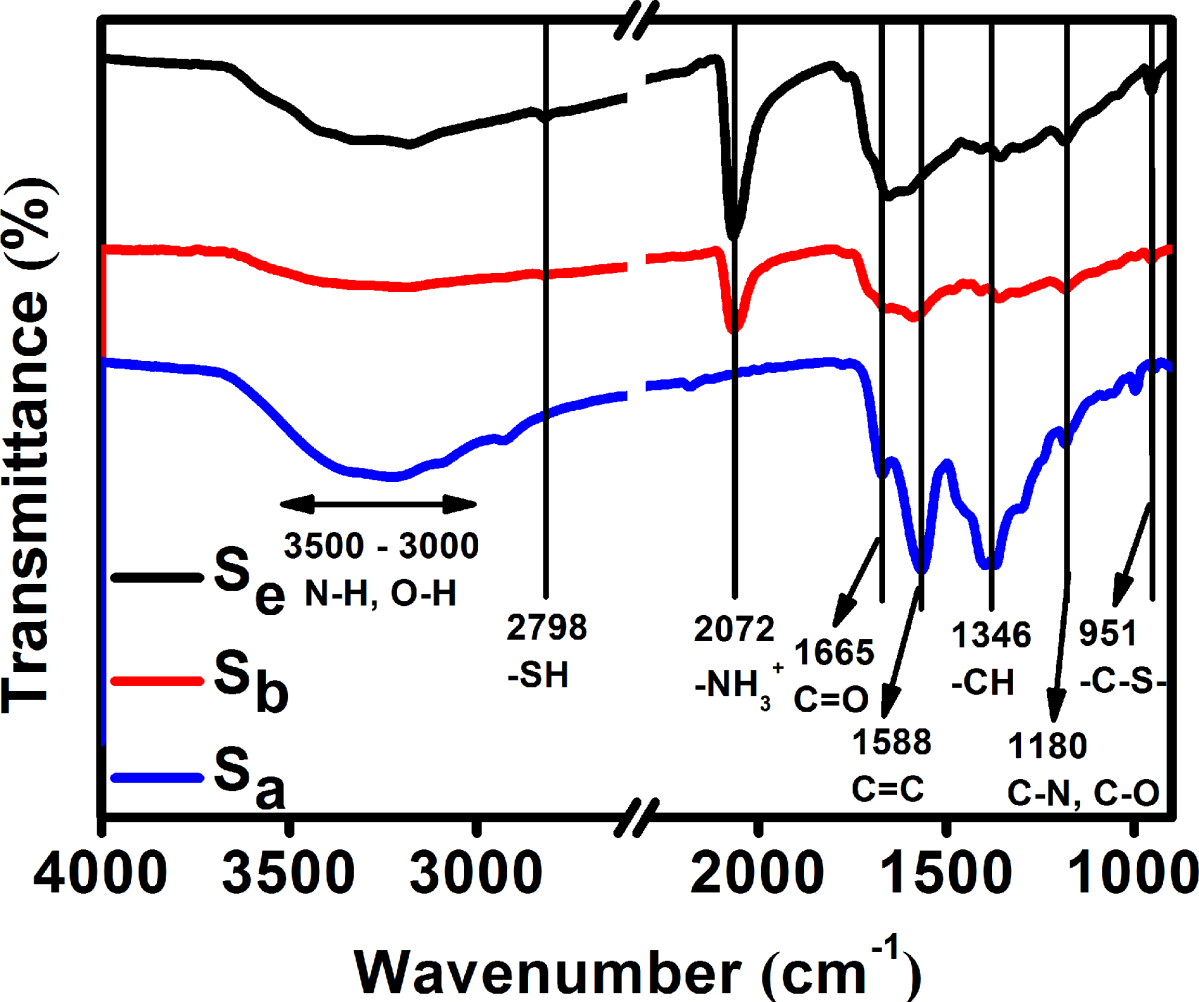
Fourier transform infrared spectra of S_a_, S_b,_ and S_e_.

The FT-IR spectra show the functional groups present in the C-dots (Figure 3). All the samples were found to contain oxygen-based functional groups (O–H, C–O, C=O), as well as N–H, C=C, and C–N groups. For S_b_ and S_e_, they have additional –NH_3_^+^ and sulphur-containing groups, such as S–H and C–S. The weak peak at 1665 cm^−1^ of S_b_ indicates an effective reduction of C=O groups.

**Figure 4:**
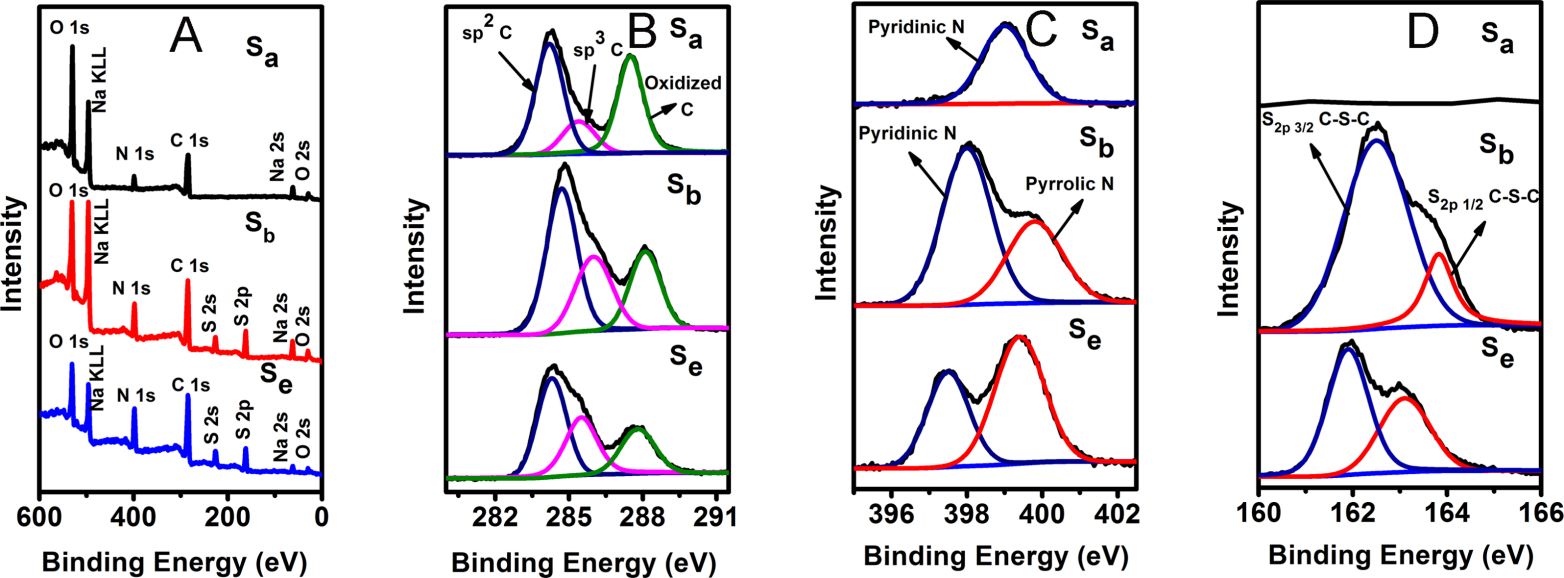
XPS spectra of S_a_, S_b,_ and S_e_. (A) full scan, (B) high-resolution C 1s XPS spectra, (C) high-resolution N 1s XPS spectra, and (D) high-resolution S 2p XPS spectra.

The chemical composition of the C-dots was further characterized by X-ray photoelectron spectroscopy (XPS) (Figure 4). The C 1s spectra can be fitted by three Gaussian peaks (Figure 4B), which correspond to the sp^2^-hybridized olefinic carbons (C=C), the sp^3^-hybridized carbons (C–O, C–S, and C–N), and the oxidized carbon in the carboxyl group. For the S_b_ sample, the intensity of carboxyl group decreases, whereas the sp^2^ C=C peak increases. Moreover, a higher binding energy of the graphitic carbon in S_b_ (284.7 eV) is found compared that of S_a_ (284.2 eV) and S_e_ (284.3 eV). Increased olefinic sp^2^ C-bond groups, with shorter bond lengths due to charge neutralization, lead to a stronger interaction between C atoms and higher binding energy. This results in a highly reduced sp^2^ structure of S_b_ [37]. The corresponding analytical outcomes are summarized in Table S3, Supporting Information File 1. Thus, S_b_ exhibits the highest sp^2^ carbon structure ratio, compared to S_a_ and S_e_. According to Figure 4C (N 1s spectra fitting), S_b_ has the highest intensity of pyridinic-N, suggesting that N atoms are more likely to form pyridinic-N structure during the co-reduction process. The detailed sample data information is presented in Supporting Information File 1, Table S4. The S 2p spectra of S_e_ and S_b_ reveal the presence of C–S–C (Figure 4D) [38–39]. Overall, XPS and FT-IR results further confirm higher conjugated sp^2^ C structures in sample S_b_, leading to an enhanced fluorescence intensity.

### Carbon nanodots as fluorescent probes for bacteria bioimaging

**Figure 5:**
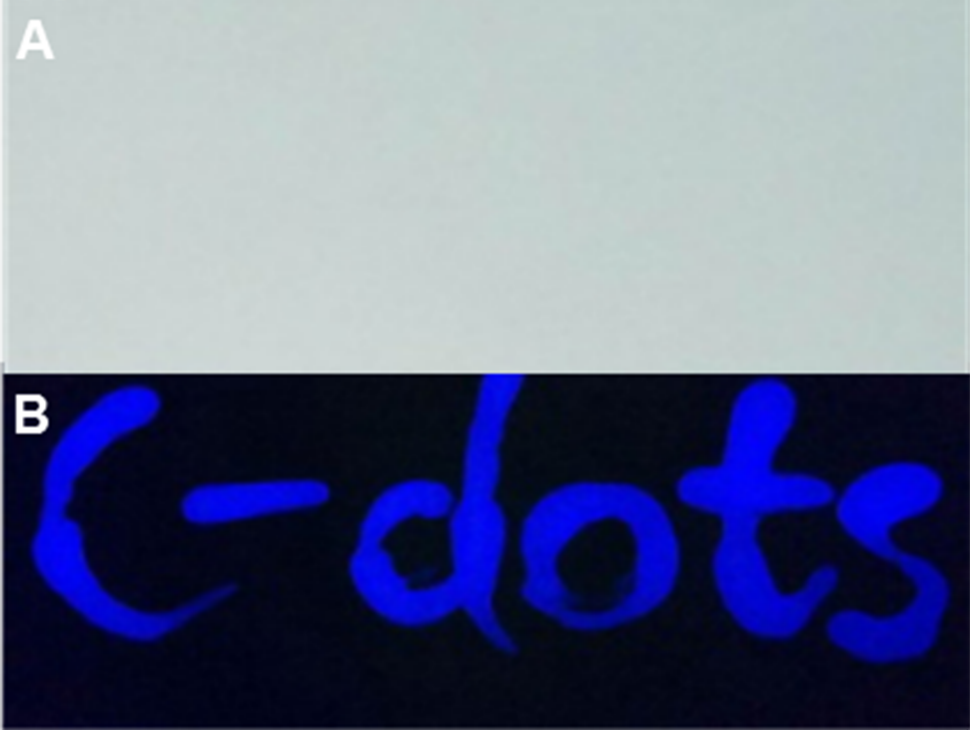
Symbols written on commercially available filter paper using S_b_ (5.0 μg mL^−1^) captured under (A) daylight and (B) UV irradiation of λ_ex_ =365 nm.

To explore the potential applications of the high QY C-dots, the highest QY C-dots, S_b_, were selected and first utilized to assess their fluorescent characteristics. As shown in Figure 5A (on commercially available filter paper), the characters cannot be detected in the visible wavelength range. Conversely, under UV excitation (λ_ex_ = 365 nm), the blue fluorescent characters “C-dots” are observed (Figure 5B).

**Figure 6:**
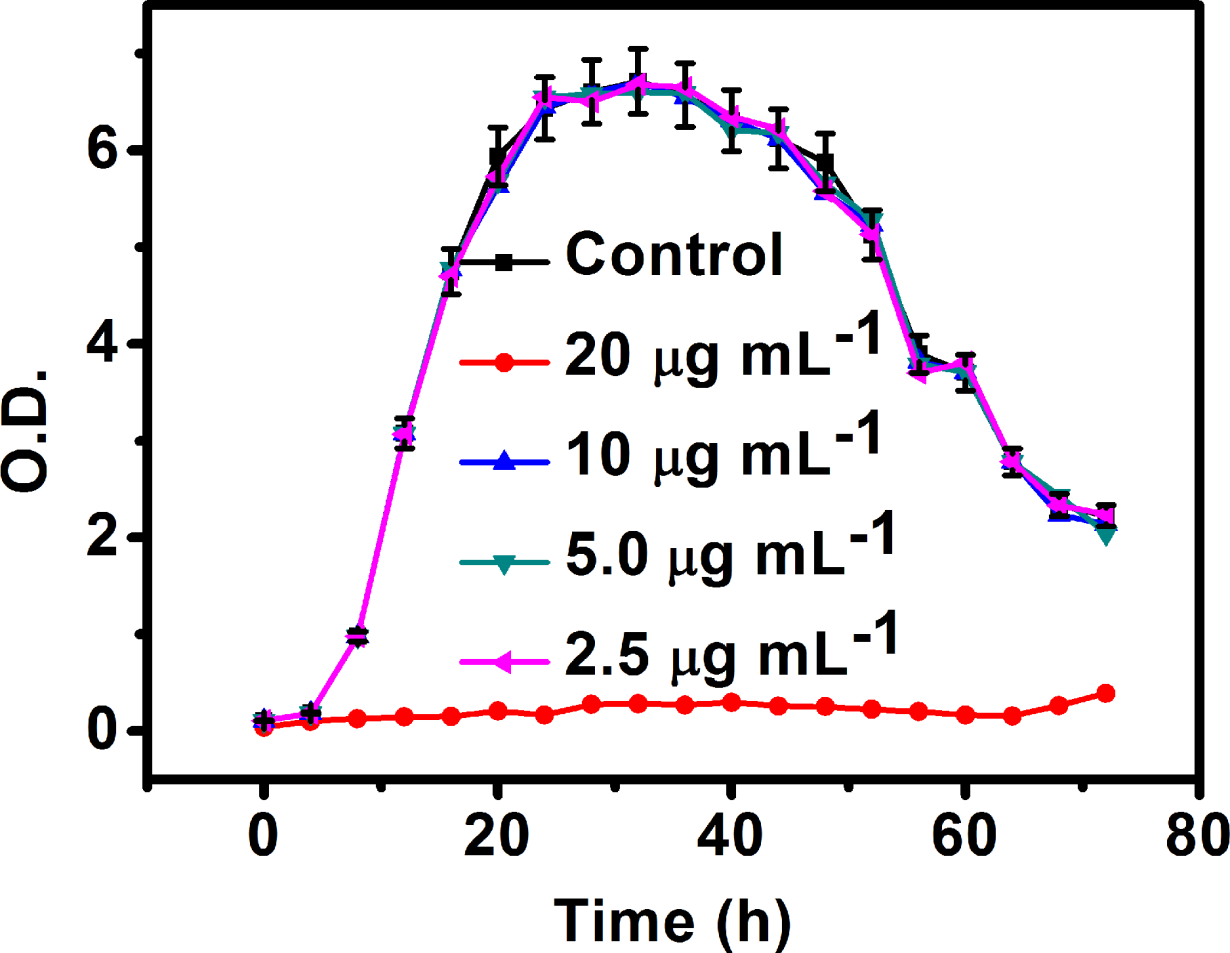
Cytotoxicity towards the bacteria *Xag* after incubation with S_b_ in the concentration range 2.5–20 µg mL^−1^ for 72 h.

After confirming the bright feature behavior, S_b_ was further used to evaluate its bioimaging properties and bacteria viability range. First of all, a cytotoxicity quantification related to the applicable C-dot concentration range was assessed. *Xag* viability was evaluated following incubation with S_b_ in the concentration range from 2.5 to 20 µg mL^−1^ for 72 h. A positive control (untreated cells) was provided, whereas different time points were designated within the 72 h interval. The data are presented in Figure 6 as the mean ± standard deviation (SD). The results show that no significant cytotoxicity is reported when the concentration of C-dots is lower than 20 µg mL^−1^. Therefore, C-dots at 20 µg mL^−1^ concentration are considered as the negative control.

**Figure 7:**
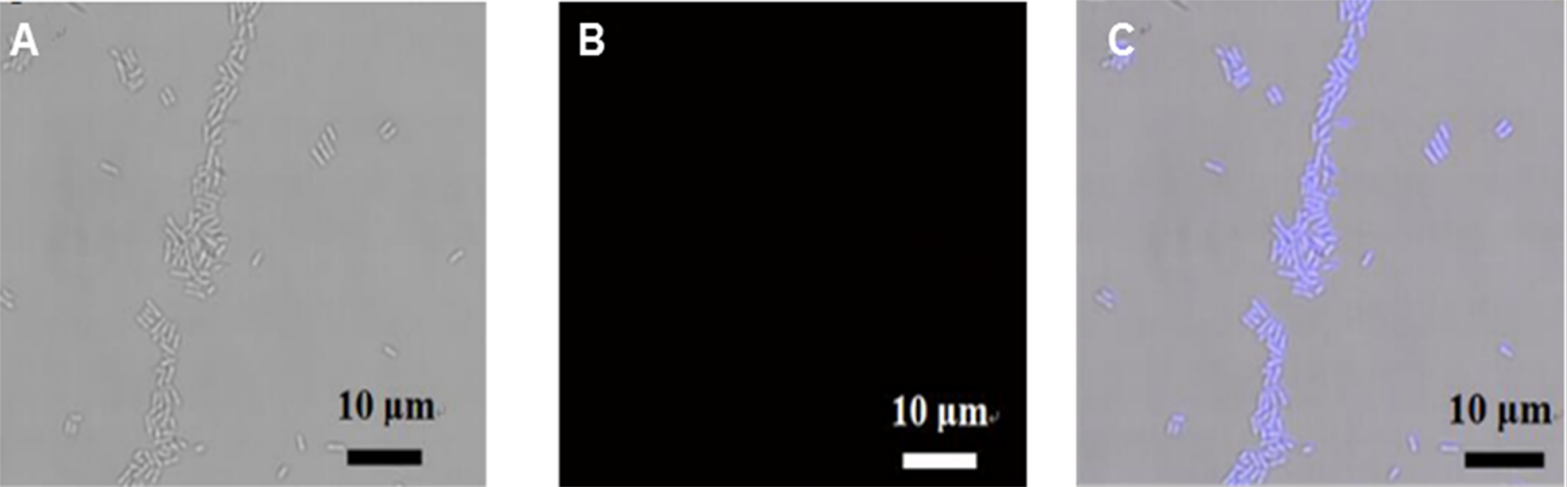
Confocal images of *Xag*. (A) Bright field without S_b_, (B) fluorescence mode without S_b_ and (C) merged channel image with 5.0 μg mL^−1^ of S_b_ incubated for 3 h at 37 °C.

Related to the C-dot assay for bioimaging applications, *Xag* bacteria were incubated and treated for confocal analysis, as detailed in the Experimental section. According to cytotoxicity assay results, *Xag* bacteria were incubated with 5.0 μg mL^−1^ of S_b_ for 3 h at 37 °C. As shown in Figure 7C, a strong blue fluorescence is observed with *λ*_ex_ = 405 nm excitation, whereas no fluorescence signal is detected from the control sample without S_b_ (Figure 7B). Moreover, both treated and untreated cells appear healthy and consistently preserved, as previously observed in cytotoxicity experiments.

## Conclusion

High QY (37%) C-dots were synthesized using a direct, simple, one-step reduction reaction process with thiourea and urea as the reducers. Remarkably, the C-dots obtained by the co-reduction showed a highly reduced sp^2^ structure, bearing a notably high QY. Compared with a conventional two-step chemical reduction pathway, the one-step method is efficient and eco-friendly. Moreover, the obtained C-dots, abundant with the hydrophilic surface, exhibited excellent fluorescent features. On the other hand, a low cytotoxicity for a model bacterial strain *Xag* was found*.* Confocal analysis confirmed the suitability of the C-dots as a bioimaging tool for a model bacterial strain, with specific optimal sample synthesis and concentration targeted at this purpose. These unique characteristics look quite promising for the employment of the described systems as fluorescent probes for bioimaging of bacteria *Xag*.

## Experimental

### Chemicals and materials

Sodium citrate, urea, and thiourea were purchased from Chemical Reagent Co. Ltd. (Tianjin, China). Xylose and glucose were purchased from Aladdin Ltd. (Shanghai, China). Nutrient broth (NB) medium (1 g L^−1^ yeast extract, 3 g L^−1^ beef extract, 5 g L^−1^ poly peptone, 10 g L^−1^ sucrose) was purchased from Sigma-Aldrich. All other chemicals were of analytical grade and used as received. Double distilled water was used in all experiments.

### Synthesis of carbon nanodots

The C-dots were synthesized by mixing different amounts of thiourea, urea or sodium citrate in 30 mL of distilled water (see Table 1 in Results and Discussion). The aqueous solutions were subsequently transferred to a 50 mL teflon-lined autoclave and heated at a constant temperature of 185 °C for 6 h. After the end of the reaction, suspensions with different turbidity were obtained, evidencing the formation of the C-dots. Then, they were dialyzed against deionized water through a dialyzer with a cut off of *M*_w_ = 1000 Da for 40 h. Finally, the resulting solutions were vacuum-evaporated in a rotary evaporator at 50 °C and then freeze-dried to obtain the the C-dot powder. The C-dots were dispersed in ultrapure water as stock solutions (0.5 mg mL^−1^) for further characterization and use. Table 1 (Results and Discussion) shows the samples with different additives obtained and labeled as S_a_, S_b_, S_c_, S_d,_ and S_e_, respectively.

### Carbon nanodot characterization

The product morphology was assessed by TEM and HRTEM, which was performed on a JEOL-2100F instrument with an accelerating voltage of 200 kV. The XRD patterns of S_a_, S_b_, and S_e_ were recorded on a Bruker D8 Advance device with a graphite monochromatized Cu Kα radiation source (λ = 1.54056 Å). XRD diagrams were recorded from 10° to 60° with a step size of 0.02° at 3° min^−1^. Raman measurements were performed with a Renishaw RM1000 confocal microscope and a He–Ne laser (633 nm, 10 mW). The laser beam was focused to a spot approximately 2 μm in diameter with a 50× microscope objective; the accumulation time was 10 s. Raman spectra were collected from several randomly selected positions on the substrate. Further evidence of the product composition was inferred by means of X-ray photoelectron spectroscopy (XPS), using a K-Alpha XPS spectrometer (Scientific Escalab 250) with an Al Kα X-ray radiation (1486.6 eV) source for excitation. UV–vis absorption spectra of the samples were recorded on a Perkin Elmer Lambda 950 spectrophotometer. FT-IR was conducted at room temperature on a Nicolet 670 spectrometer in the range of 4000–400 cm^−1^. An LS-45 fluorescence spectrophotometer (Perkin-Elmer, UK) was employed for fluorescence spectroscopy measurements. Confocal images were acquired using a confocal laser scanning microscope (Leica TCS SP5 AOBS).

### Fluorescence related experiments

The relative quantum yield was measured according to the equation [40]:

[1]
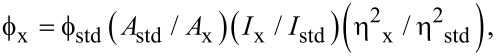


where Φ is the quantum yield, *A* is the optical density, *I* is the measured integrated emission intensity, and η is the refractive index. The subscript "std" indicates the value of a standard reference and "x" for the sample. Quinine sulfate (Φ_std_ = 0.54) was used as the standard and was dissolved in 0.1 M H_2_SO_4_ (η_std_ = 1.33) and the C-dots were dissolved in water (η_x_ = 1.33). In order to minimize re-absorption effects, absorbance readings in a 10 mm fluorescence cuvette were kept near 0.05 at the excitation wavelength (λ_ex_ = 360 nm).

Fluorescence lifetime parameters were monitored at room temperature in aqueous solution by using the time-correlated single-photon counting system in the FLS980 device. An Edinburgh EPL 340 ps pulsed diode laser (341.6 nm, 701.2 ps pulse width) operated at 200 kHz was used as the excitation source. The fluorescence lifetime (τ) was fitted by using the Edinburgh FLS980 software package. The average lifetime (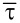) was calculated according to the following equation

[2]
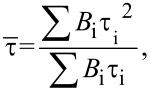


where *B*_i_ is the fractional contribution of the time-resolved decay lifetime of τ_i_.

To further prove and demonstrate the fluorescent features of the C-dots, the colorless aqueous solution of the C-dots (S_b_, 5.0 μg mL^−1^) was applied to commercial filter paper using a Chinese brush. The abbreviation “C-dots” was observed under UV lamp excitation (λ_ex_ = 365 nm).

### Confocal microscopic imaging of bacteria using carbon nanodots as bioimaging probes

*Xanthomonas axonopodis pv. glycins* (*Xag*) strains were used as the bacterial model. *Xag* were grown in NB medium at 28 °C for 24 h. 200 μL of *Xag* grown in NB medium was then inoculated into 20 mL of fresh medium and grown in a shaking incubator (200 rpm) at 28 °C for 18 h. Then, 5 mL of the bacteria in the middle of an exponential growth phase were collected by centrifugation at 3000 rpm for 10 min (20 °C) and fixed in 1 mL 70% ethanol for 5 min at 4 °C. The fixed bacteria were suspended in NB medium containing C-dots (5.0 μg mL^−1^) while shaking for 3 h at 28 °C. The final bacteria pellets were washed with PBS, resuspended in 200 μL of PBS, and then further transferred to a glass slide for confocal imaging using an excitation wavelength of 405 nm. All images were acquired at 630× magnification.

### Cytotoxicity assay of carbon nanodots for *Xag*

The cytotoxicity of C-dots toward *Xag* was measured in NB medium at 28 °C. Different concentrations of C-dots (0, 2.5, 5.0, 10 and 20 μg mL^−1^) were added into Erlenmeyer flasks and shaken for 2 min at 180 rpm. Furthermore, 0.2 mL of culture broth was collected at different time points (0–72 h) and their optical density was measured at 600 nm in order to calculate the cell viability. Quantification is reported as relative values to the negative control, where the negative control (untreated) is set to 100% viability.

## Supporting Information

File 1 Additional experimental data.
The general co-reduction method with urea and thiourea to obtain C-dots, the scale-up synthesis of C-dots, TEM and size distribution of S_b50_, XRD patterns of S_a_, S_b_ and S_e_, Raman spectra of S_a_, S_b_ and S_e_, deconvoluted C 1s XPS spectra of different C-dots with peak area (*A*) ratios of the sp^3^ C or oxidized C to the sp^2^ C and deconvoluted N 1s XPS spectra of different C-dots with *A* ratios of the pyrrolic N to pyridinic N.
