# Supplementary material for: Co-reductive fabrication of carbon nanodots with high quantum yield for bioimaging of bacteria
Source: Beilstein J Nanotechnol. 2018 Jan 12;9:137–45. doi: 10.3762/bjnano.9.16 (PMC5789424; doi:10.3762/bjnano.9.16)
Supplement: File 1 — Additional experimental data. The general co-reduction method with urea and thiourea to obtain C-dots, the scale-up synthesis of C-dots, TEM and size distribution of Sb50, XRD patterns of Sa, Sb and Se, Raman spectra of Sa, Sb and Se, deconvoluted C 1s XPS spectra of different C-dots with peak area (A) ratios of the sp3 C or oxidized C to the sp2 C and deconvoluted N 1s XPS spectra of different C-dots with A ratios of the pyrrolic N to pyridinic N. [file Beilstein_J_Nanotechnol-09-137-s001.pdf]

Supporting Information

for

Co-reductive fabrication of carbon nanodots with high quantum yield for bioimaging of bacteria

Jiajun Wang[1], Xia Liu[1], Gesmi Milcovich[2], Tzu-Yu Chen[3], Edel Durack[2], Sarah Mallen[2], Yongming Ruan[1], Xuexiang Weng*[1] and Sarah P. Hudson*[2]

Address: [1]College of Chemistry and Life Science, Zhejiang Normal University, Jinhua 321004, PR China, [2]Department of Chemical Sciences, Bernal Institute, University of Limerick, Castletroy, Ireland and [3]School of Chemistry, University of Birmingham, Edgbaston, Birmingham B15 2TT, UK

Email: Xuexiang Weng - xuexian@zjnu.cn; Sarah P. Hudson - sarah.hudson@ul.ie

* Corresponding author

Additional experimental data

Content

1. The generality of co-reduction method with urea and thiourea to obtain C-dots (Table S1).
2. The scale-up synthesis of C-dots (Table S2).
3. TEM and size distribution of S_{b50} (Figure S1).
4. XRD patterns of $S_{a,}$ $S_{b\ and}$ S_e (Figure S2).
5. Raman spectra of S_a, S_b and S_e (Figure S3).
6. Deconvoluted C1s XPS spectra of different C-dots with peak area (*A*) ratios of the sp^3 C or oxidized C to the sp^2 C (Table S3).
7. Deconvoluted N1s XPS spectra of different C-dots with *A* ratios of the pyrrolic N to pyridinic N (Table S4).

Table S1: The general co-reduction method with urea and thiourea to obtain C-dots.

Serial[a]	Different carbon sources (mmol)	Urea (mmol)	Thiourea (mmol)	QY (%)
S1	Glucose (0.28)	1.26	0.42	16
S1U	Glucose (0.28)	1.68	0	3
S1T	Glucose (0.28)	0	1.69	2
S2	Xylose (0.28)	1.26	0.42	14
S2U	Xylose (0.28)	1.68	0	1
S2T	Xylose (0.28)	0	1.69	2

[a]All the samples were dissolved in 30 mL of water.

Table S2: The scale-up synthesis of C-dots.

Serial[a]	Sodium citrate (mmol)	Urea (mmol)	Thiourea (mmol)	Quantitative yield (g)	QY (%)
S_{a50}	14.0	84.0	0	3.08	10
S_{b50}	14.0	63.0	21.0	5.95	25
S_{e50}	14.0	0	84.0	4.85	2

[a]All the samples were dissolved in 30 mL of water.

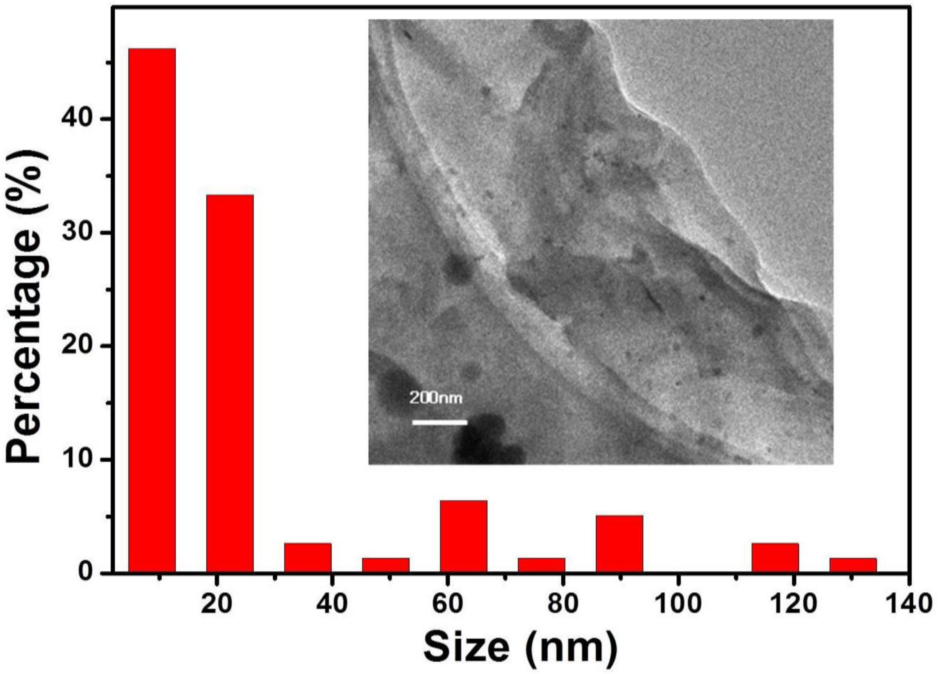

Figure S1: The TEM image (inset) and size distribution of S_{b50}.

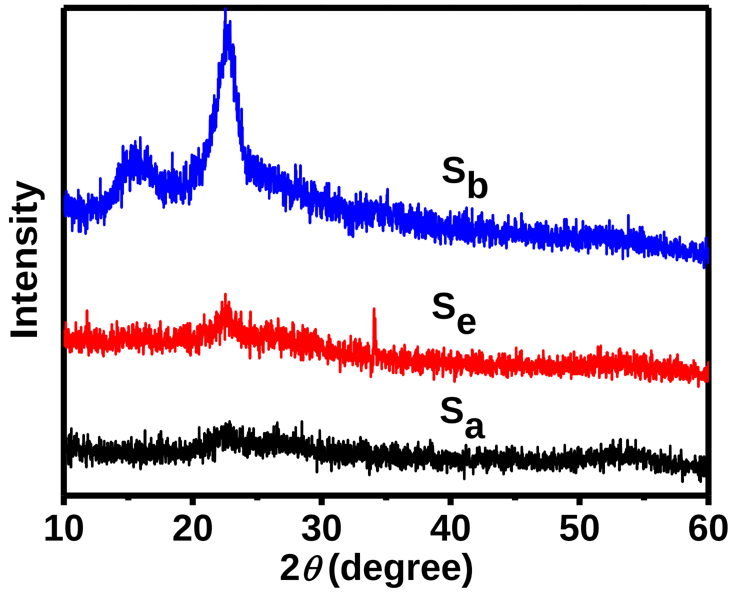

Figure S2: XRD patterns of S_a, S_b and S_e.

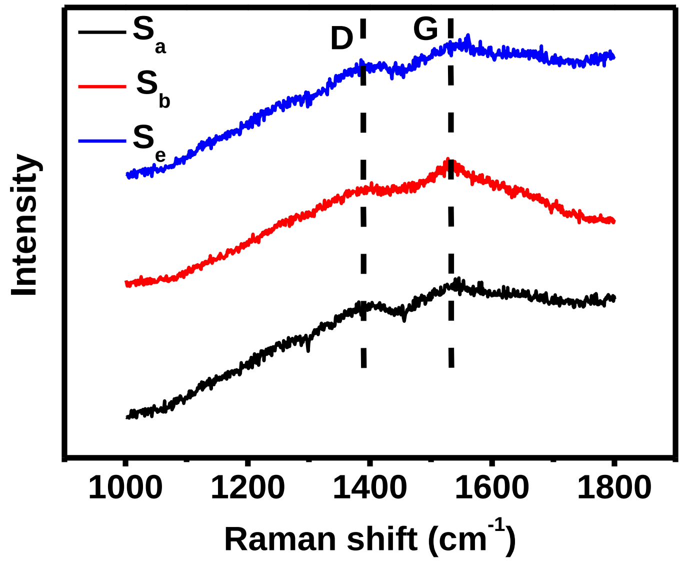

Figure S3: Raman spectra of S_a, S_b and S_e.

Table S3: Detailed information of the deconvoluted C 1s XPS spectra of different C-dots with peak area (*A*) ratios of the sp^3 C or oxidized C to the sp^2 C.

Sample	Band	sp^2 C	sp^3 C	Oxidized C
S_a	Position/eV	284.2	285.4	287.5
	Area	16934.0	5557.4	15158.3
	$A/A_{sp^2\ C}$	1	0.328	0.895
S_b	Position/eV	284.7	286.0	288.1
	Area	24323.5	14305.8	12436.8
	$A/A_{sp^2\ C}$	1	0.588	0.511
S_e	Position/eV	284.3	285.5	287.8
	Area	15281.0	11066.1	8056.7
	$A/A_{sp^2\ C}$	1	0.724	0.527

Table S4: Detailed information of the deconvoluted N 1s XPS spectra of different C-dots with peak area (A) ratios of the pyrrolic N to pyridinic N.

Sample	Band	Pyrrolic N	Pyridinic N
S_a	Position/eV	-	399.0
	Area	-	7441.4
	$A/A_{pyridinic\ N}$	0	1
S_b	Position/eV	399.8	398.0
	Area	9550.8	15273.3
	$A/A_{pyridinic\ N}$	0.625	1
S_e	Position/eV	399.4	397.5
	Area	12495.6	9216.9
	$A/A_{pyridinic\ N}$	1.356	1
